# Differential effects on acetaminophen-induced nephrotoxicity and liver injury following modulation of glutathione resynthesis

**DOI:** 10.1016/j.fct.2025.115896

**Published:** 2025-12-09

**Authors:** Yasaman Etemadi, Jephte Y. Akakpo, Timothy A. Fields, Anup Ramachandran, Hartmut Jaeschke

**Affiliations:** a Department of Pharmacology, Toxicology, and Therapeutics, University of Kansas Medical Center, Kansas City, KS, 66160, USA; b Department of Pathology and Laboratory Medicine, University of Kansas Medical Center, Kansas City, KS, 66160, USA

**Keywords:** Acute kidney injury, Acute liver failure, Acetaminophen, N-acetylcysteine, Buthionine sulfoximine, Glutathione

## Abstract

Acetaminophen (APAP) overdose is a leading cause of acute liver failure (ALF), with acute kidney injury (AKI) increasing morbidity and mortality. N-acetylcysteine (NAC) prevents APAP-induced liver damage, but not AKI, highlighting the need to address differential inter-organ responses to APAP toxicity. We investigated the relationship between hepatic glutathione (GSH) depletion, liver injury, and subsequent kidney damage following APAP overdose. Male C57BL/6J mice received either moderate (300 mg/kg) or severe (600 mg/kg) overdoses of APAP, with or without buthionine sulfoximine (BSO, 50 mg/kg) to deplete GSH, or NAC (500 mg/kg) to replenish GSH. A moderate APAP overdose elevated liver injury markers (alanine aminotransferase, ALT) without significantly affecting blood urea nitrogen (BUN) levels, though kidney injury molecule-1 (KIM-1) expression increased. A severe overdose significantly increased ALT activities, and BUN and creatine levels, together with marked upregulation of renal KIM-1 and histological evidence of cortical damage. BSO exacerbated APAP-induced kidney but not liver injury, where GSH remained depleted at 24 h. In contrast, NAC protected against APAP hepatotoxicity but not AKI. Thus, these findings demonstrate critical organ-specific responses to APAP toxicity and underscore the need for targeted therapeutic strategies specifically addressing APAP-induced kidney injury.

## 1. Introduction

Acetaminophen (APAP) overdose is a primary cause of acute liver failure (ALF), particularly in Western countries. Notably, APAP is responsible for 46 % of all ALF cases in the United States (Hodgman and Garrard, 2012; Ostapowicz et al., 2002). At therapeutic doses, APAP is mainly metabolized by the liver through glucuronidation and sulfation. A small fraction, however, is bioactivated by the cytochrome P450 enzyme CYP2E1 to form the reactive metabolite N-acetyl-*p*-benzoquinone imine (NAPQI) (Jaeschke and Ramachandran, 2024; McGill and Jaeschke, 2013). Under normal conditions, NAPQI is detoxified through conjugation with glutathione (GSH), a tripeptide composed of glutamate, cysteine, and glycine (McGill and Jaeschke, 2013; Meister, 1988). However, in overdose situations, excessive NAPQI depletes hepatic GSH levels and covalently binds to cellular proteins (Mitchell et al., 1973), leading to mitochondrial dysfunction and necrotic cell death (Gujral et al., 2002; Jaeschke et al., 2019; Ramachandran and Jaeschke, 2019).

While the liver is the primary target of APAP toxicity, growing evidence suggests that APAP overdose also significantly increases the risk of acute kidney injury (AKI) (Tujios et al., 2015). Several previous studies have reported a substantial prevalence of AKI in APAP overdose patients, especially in those with severe liver injury (Akakpo et al., 2020; Antoine et al., 2015; Mour et al., 2005; Stollings et al., 2016; Tujios et al., 2015), highlighting the increasing recognition of kidney complications in APAP toxicity. Supporting this clinical significance, our previous analysis of plasma creatinine levels and liver enzymes in APAP overdose patients demonstrated a clear relationship between liver and kidney injury. Specifically, our findings revealed that all patients who developed renal failure exhibited significantly more severe liver injury than those who did not (Akakpo et al., 2020). The clinical importance of this liver-kidney connection is further underscored by observations that AKI generally worsens the clinical prognosis in both APAP and Non-APAP ALF (Antoine et al., 2015; Campbell and Baylis, 1992; Hadem et al., 2019; Leithead et al., 2009). KIM-1 levels were substantially higher in APAP overdose patients with poor outcomes, suggesting that low KIM-1 may be a prognostic biomarker for survival (Antoine et al., 2015). Furthermore, patients who developed AKI experienced significantly lower one-year survival rates compared to those without kidney injury (Tujios et al., 2015). Thus, AKI is a significant contributor to the overall outcome in APAP overdose patients.

Although Cyp2E1 plays a crucial role in APAP metabolism and NAPQI formation in both hepatocytes and proximal tubular cells of the kidney (Akakpo et al., 2020; Hart et al., 1994, 1995), growing evidence, including our previous work, indicates that early mechanisms of APAP-induced nephrotoxicity are distinct from those involved in hepatotoxicity (Akakpo et al., 2020, 2023; Eguia and Materson, 1997; Jones and Vale, 1993; Mour et al., 2005). In the kidneys, APAP-induced toxicity primarily involves endoplasmic reticulum (ER) stress and caspase-mediated apoptosis, rather than mitochondrial dysfunction (Akakpo et al., 2023; Lorz et al., 2004). Notably, we have shown that the caspase inhibitor Ac-DEVD-CHO can alleviate ER stress and prevent kidney damage without affecting liver injury, highlighting the organ-specific nature of APAP toxicity (Akakpo et al., 2023). This organ specificity is further demonstrated by the therapeutic limitations of N-acetylcysteine (NAC), the only clinically approved antidote for APAP toxicity. While NAC effectively protects against APAP-induced hepatotoxicity by restoring depleted glutathione (GSH) in the liver and scavenging NAPQI and peroxynitrite (Corcoran et al., 1985; Saito et al., 2010), endogenous GSH-derived APAP metabolites have been implicated in APAP-induced kidney injury (Emeigh-Hart et al., 1996). In addition, APAP-cysteine pretreatment enhanced APAP-induced nephrotoxicity (Stern et al., 2005a, 2005b). Further experiments demonstrated that NAC, administered orally or intraperitoneally, did not protect against APAP-induced kidney injury (Slitt et al., 2004). These findings correlated with case reports indicating that NAC did not affect nephrotoxicity in APAP overdose patients (Davenport and Finn, 1988; Hengy et al., 2009; Jones and Vale, 1993).

The relationship between APAP-induced liver and kidney injury is complex and not fully understood. Some studies suggest a potential liver-kidney crosstalk, with evidence of liver injury influencing kidney Cyp2E1 expression through microRNA-mediated pathways (Matthews et al., 2020). However, several clinical reports support the hypothesis that APAP-induced nephrotoxicity can occur independently of hepatotoxicity (Campbell and Baylis, 1992; Hadem et al., 2019; Leithead et al., 2009). These findings highlight the complexity of APAP toxicity and underscore the importance of considering organ-specific mechanisms when developing therapeutic strategies.

Given these organ-specific differences and the critical role of GSH in APAP metabolism, we utilized l-buthionine-S, R-sulfoximine (BSO), a specific inhibitor of γ-glutamyl-cysteine synthetase, the key enzyme in GSH synthesis, to deplete cellular GSH (Drew and Miners, 1984; Meister, 1988). In addition, we cotreated with NAC to promote GSH synthesis. These approaches enabled us to examine the impact of GSH depletion and improved recovery in the liver and kidney, and its effect on the respective organ injury by an APAP overdose. The objective was to assess whether manipulation of organ GSH levels would modulate liver and kidney injury similarly or differently. In the latter case, it would provide evidence for the independence of the injury mechanisms in both organs.

## 2. Materials and methods

### 2.1. Animals

8–10-week-old male C57BL/6J mice were obtained from Jackson Laboratories (Bar Harbor, Maine, USA) and housed under standard conditions with free access to food and water. Mice were housed in a temperature- and light-controlled facility with a 12-h light-dark cycle. All experiments and animal procedures were reviewed and approved by the Institutional Animal Care and Use Committee at the University of Kansas Medical Center to ensure ethical standards consistent with National Research Council guidelines for laboratory animal welfare were met. All procedures performed in the study involving animals complied with the ARRIVE guidelines.

### 2.2. Experimental design

Reagents were purchased from Sigma-Aldrich (St. Louis, Missouri) unless otherwise stated. Mice were fasted for 16 h overnight before treatment. APAP was dissolved in warm saline and administered via intraperitoneal (i.p.) injection at doses of either 300 or 600 mg/kg. For experiments with BSO, mice were treated with BSO (50 mg/kg in saline, i. p.) alone or 2 h before receiving APAP (300 mg/kg, i. p.). For experiments with NAC, mice were administered NAC (500 mg/kg, i. p.) alone or concurrently with APAP (600 mg/kg, i. p.). After 3, 6, or 24 h, mice were euthanized under isoflurane anesthesia. Blood was immediately collected from the inferior vena cava using a heparinized syringe and centrifuged at 18,000 g for 2 min to isolate plasma. Livers and kidneys were harvested, rinsed in saline, and processed. Some tissue sections were fixed in 10 % phosphate-buffered formalin overnight for histological analysis. The remaining liver tissue was flash frozen in liquid nitrogen for molecular studies.

### 2.3. Biochemical assays

Plasma levels of the hepatic enzyme alanine aminotransferase (ALT) were quantified using a commercial colorimetric assay kit (MedTest, Canton, MI, USA), carried out according to the manufacturer’s standardized protocol. To evaluate the severity of kidney dysfunction, blood urea nitrogen (BUN) levels were determined using a QuantiChrom^™^ Urea Assay kit from BioAssay Systems (Hayward, CA). Creatinine levels were determined using a QuantiChrom^™^ creatinine Assay kit from BioAssay Systems (Hayward, CA). Tissue GSH levels were assayed with a modified Tietze assay as described in detail (McGill and Jaeschke, 2015).

### 2.4. Histology and immunohistochemistry

For histological analysis, liver and kidney tissues were fixed in neutral buffered formalin solution before undergoing paraffin embedding. Embedded tissue blocks were sectioned at 5 μm in thickness using a microtome. To assess morphological features and quantify necrotic regions, mounted tissue sections were stained with hematoxylin and eosin (H&E) or Periodic Acid–Schiff (PAS) staining according to standard protocols. AKI was scored blinded by the pathologist using the following scoring criteria based on injury severity and affected tissue percentage for each group: Score 1: Mild injury affecting <10 % of the cortex; Score 2: Mild injury affecting >50 % of the cortex; Score 3: Moderate-severe injury affecting <10 % of the cortex; Score 4: Moderate-severe injury affecting >50 % of the cortex. DNA fragmentation in liver and kidney sections was assessed using terminal deoxynucleotidyl transferase-mediated dUTP nick end-labeling (TUNEL) staining. The staining was performed with the In-Situ Cell Death Detection Kit-fluorescein (Roche Diagnostics, Indianapolis, IN; Cat#11684795910). For kidney injury molecule 1 (KIM-1) immunostaining, kidney sections were first blocked for 1 h at room temperature with 3 % BSA in serum and then incubated overnight with rabbit anti-KIM-1 antibodies (Cell Signaling Technology, #14971). To quench endogenous peroxidase activity, sections were treated with 3 % hydrogen peroxide, followed by three washes in TBS (5 min each). Signal detection was carried out using an avidin-biotin conjugate detection system (Vector Laboratories, PK-4001) followed by a diamond-benzidine (DAB) substrate kit (Cell Signaling Technology, #8059).

### 2.5. RNA isolation and quantitative PCR

Total RNA was isolated from liver and kidney tissues using TRIZOL reagent (Invitrogen) per the manufacturer’s protocol. Two micrograms of purified RNA were reverse transcribed into cDNA using a commercially available kit (Applied Biosystems #4368814). Quantitative PCR was performed using PowerUp SYBR Green Master Mix (ThermoFisher #A25742) with gene-specific primers purchased from Integrated DNA Technologies. The mRNA expression levels of neutrophil gelatinase-associated lipocalin (NGAL) and KIM-1 were quantified using the comparative CT method, with 18 S rRNA as an internal reference gene. Relative fold-changes in gene expression were calculated using the 2−ΔΔCT method.

### 2.6. Quantitation of APAP protein adducts measurements

Protein adducts were analyzed following the methods described previously (Etemadi et al., 2023). In brief, kidney tissue homogenates were filtered using Bio-Spin 6 columns (Bio-Rad, Hercules, CA) to eliminate low molecular weight metabolites that could interfere with the detection of APAP protein adducts. Tissue proteins were precipitated and then digested with proteases. After additional centrifugation, the isolated APAP-CYS residues in the supernatant were analyzed via high-pressure liquid chromatography (HPLC) equipped with a Coularray electrochemical detector (ESA Biosciences, Chelmsford, MA).

### 2.7. Statistical analysis

Quantitative data are presented as mean ± standard error of the mean (SEM). Statistical comparisons between two experimental groups were conducted using an unpaired Student’s T-test. Differences with a p-value less than 0.05 were considered to be statistically significant. All graphical representations and analyses were performed using GraphPad Prism version 8.0.1 (GraphPad Software, San Diego, CA).

## 3. Results

### 3.1. Only a severe overdose of APAP causes acute kidney injury

**Fig. 1.**
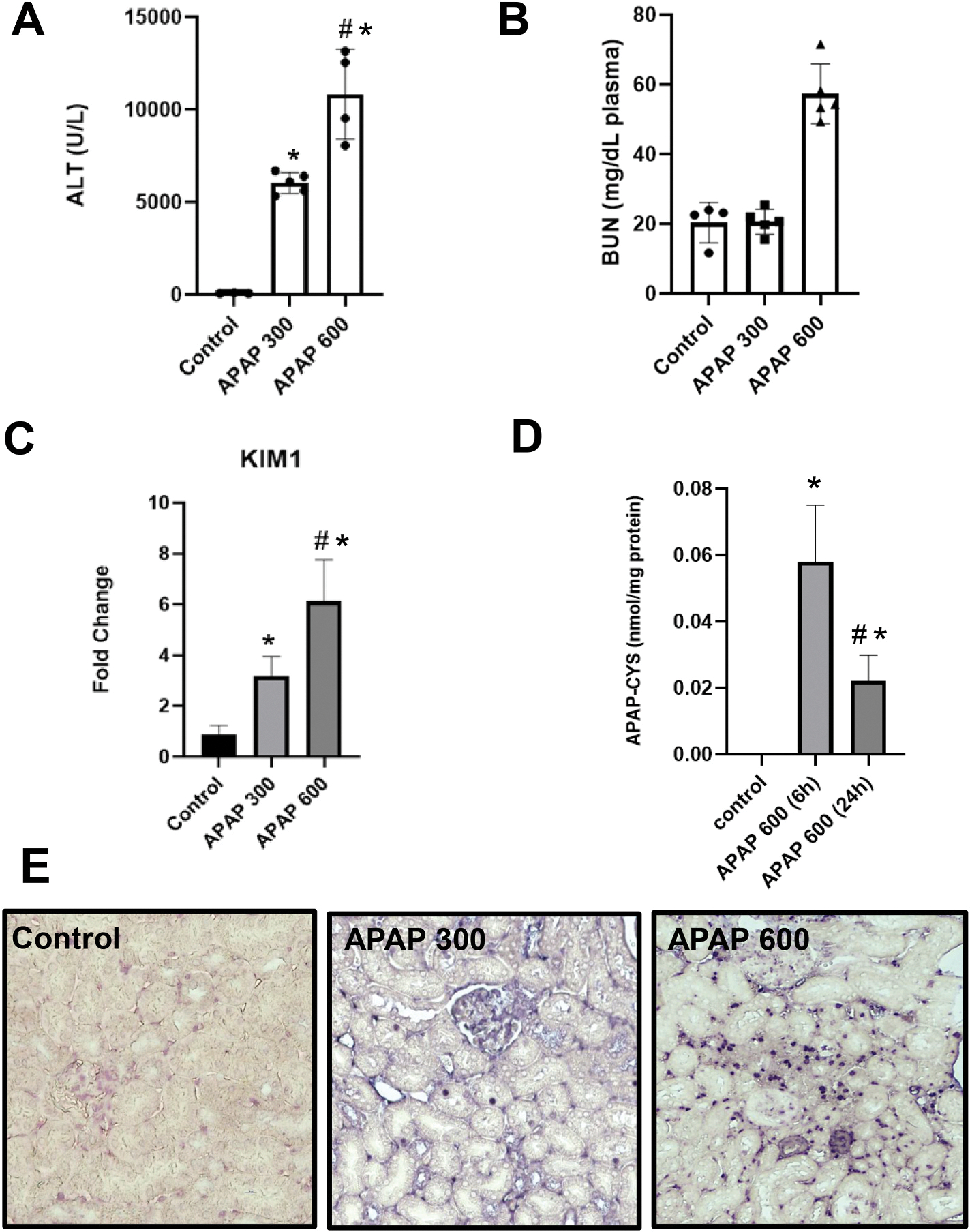
A severe overdose of APAP induces liver and acute kidney injury. Fasted male C57BL/6J mice received a single intraperitoneal injection of APAP (300 or 600 mg/kg) and were sacrificed 24 h after APAP. (A) Plasma ALT activities, (B) plasma BUN levels, (C) gene expression level of KIM-1, (D) renal APAP-protein adducts, (E) TUNEL staining (40X). Bars represent means ± SEM for n = 5 mice. *p < 0.05 compared to control; ^#^p < 0.05 compared to APAP-treated groups.

To compare dose-dependent effects of APAP on liver and kidney function in male C57BL/6J mice, animals were fasted overnight and injected intraperitoneally with either 300 or 600 mg/kg of APAP. ALT levels, a marker of liver injury, were significantly elevated in both APAP-treated groups compared to controls 24 h after APAP (Fig. 1A). Notably, BUN levels, an indicator of kidney dysfunction, significantly increased only in the 600 mg/kg APAP group (Fig. 1B). Creatinine levels did not change compared to controls after 300 mg/kg APAP (data not shown) and data for a 600 mg/kg group are shown in Fig. 5. This suggests that while APAP at 300 mg/kg induces liver injury, it does not cause significant kidney dysfunction. To further examine kidney injury, we assessed the gene expression of KIM-1, a highly sensitive and specific biomarker of AKI (Tanase et al., 2019). KIM-1 mRNA expression was elevated by 6-fold in the 600 mg/kg APAP group compared to controls, while expression in the 300 mg/kg group increased up to 3-fold compared to controls (Fig. 1C). Since the formation of APAP-protein adducts is a well-recognized hallmark of APAP-induced liver and kidney injury (Akakpo et al., 2024; McGill et al., 2013; Streeter et al., 1984), we investigated the presence of these adducts in kidney tissue to explore their role in nephrotoxicity. Given the clear evidence of renal injury at the 600 mg/kg APAP dose, this dose was selected for further evaluation. Kidney homogenates from mice receiving 600 mg/kg APAP exhibited a marked elevation in APAP-protein adduct formation, with levels peaking at 6 h post-administration before declining by 24 h (Fig. 1D).

**Fig. 2.**
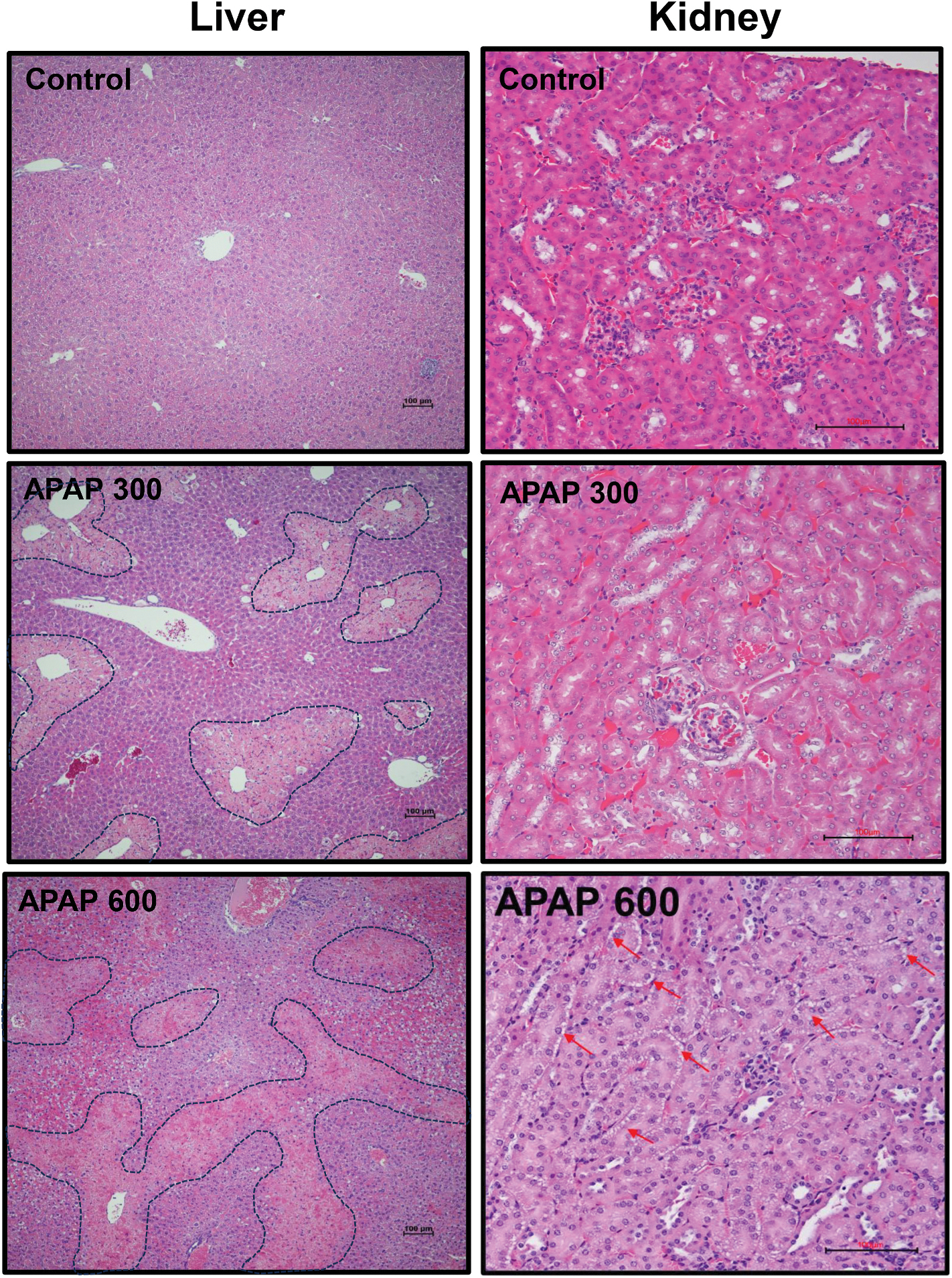
Acute liver and kidney tissue injury by a severe APAP overdose. Fasted male C57BL/6J mice received a single intraperitoneal injection of APAP (300 or 600 mg/kg) and were sacrificed 24 h after APAP. Tissue sections were stained with H&E to examine liver and kidney morphology. Representative images of liver sections (left panels) show normal hepatocytes in the control group. In contrast, liver tissue from APAP-treated mice exhibited centrilobular necrosis of hepatocytes, indicated by a dashed line. Representative kidney sections (right panels) from the control group and APAP 300 mg/kg display normal renal glomeruli and Bowman’s capsules, surrounded by intact tubules and a few vacuolar changes. In contrast, kidney tissue from mice treated with APAP 600 mg/kg showed mild injury with prominent vacuolar changes throughout the cortex (red arrows). These images are representative of five animals per group. (For interpretation of the references to color in this figure legend, the reader is referred to the Web version of this article.)

Additionally, TUNEL staining was performed to assess cell death in renal tissue. The results revealed detectable apoptotic cell death in proximal tubular cells of mice treated with 600 mg/kg APAP, highlighting significant renal cellular injury (Fig. 1E). Although a small percentage of apoptotic cells was observed in the 300 mg/kg group, apoptosis was more pronounced at the higher dose, supporting the idea that APAP-induced renal toxicity is dependent on the severity of APAP overdose (Fig. 1E). Histological analysis of liver and kidney sections confirmed the dose-dependent renal injury findings. Liver sections showed a significant increase in necrotic areas as the APAP dose increased from 300 mg/kg to 600 mg/kg (Fig. 2). This dose-dependent hepatic injury aligns with the biochemical markers of liver damage observed in Fig. 1A. Histological analysis of kidney sections from mice exposed to 300 mg/kg APAP revealed no significant differences from the control group (Fig. 2). However, in the 600 mg/kg APAP-treated group, there was clear evidence of renal injury, characterized by frequent vacuoles in the cytoplasm of proximal tubular cells (Fig. 2).

### 3.2. GSH depletion with BSO amplified APAP-induced kidney injury

**Fig. 3.**
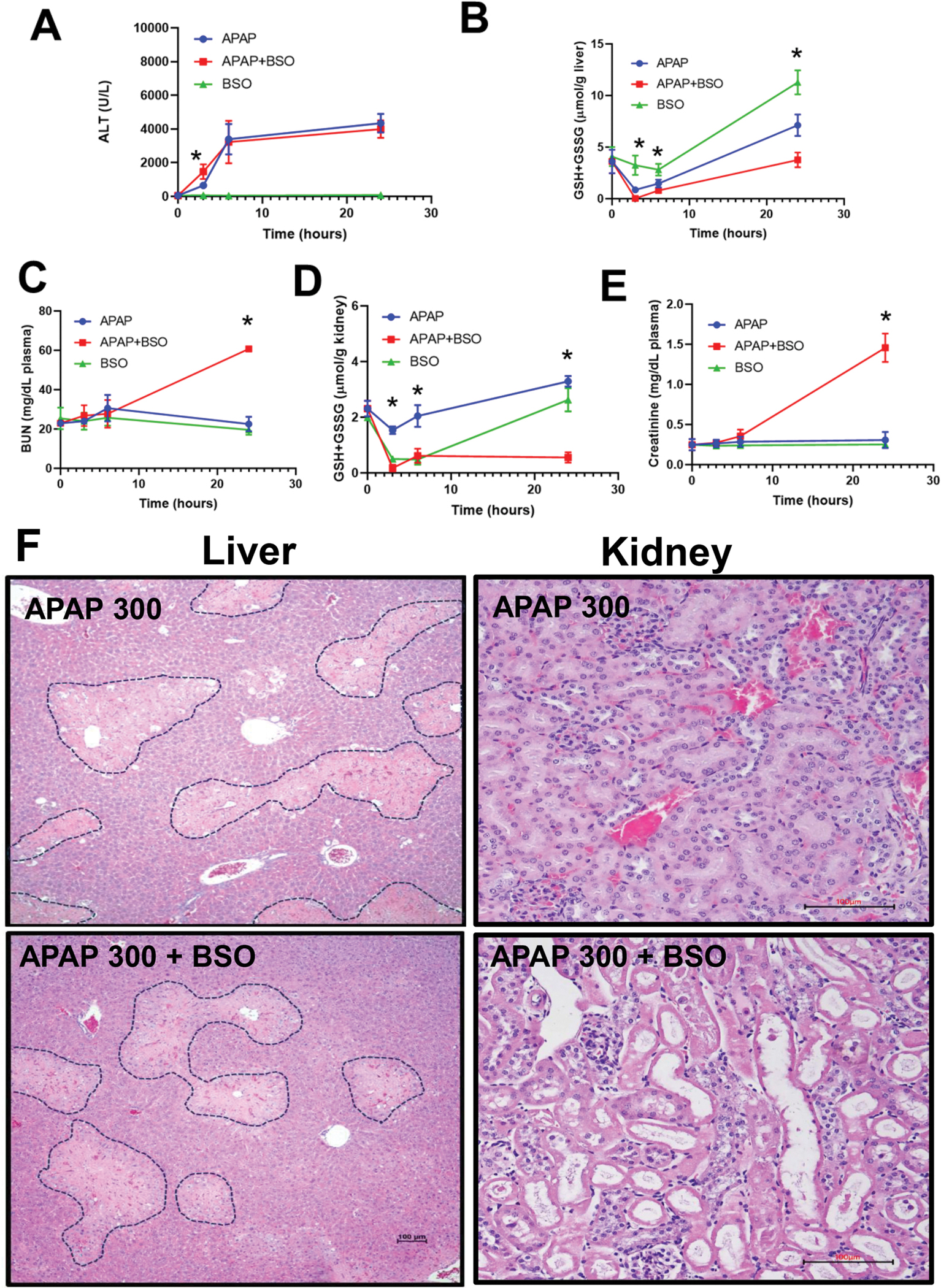
Differential impact on APAP-induced liver and kidney injury by an inhibitor of GSH synthesis. Fasted male C57BL/6J mice received a single intraperitoneal injection of APAP (300 mg/kg), either with or without a 2-h pretreatment with 50 mg/kg BSO, or BSO alone; the animals were sacrificed at 3, 6, or 24 h after APAP. (A) Plasma ALT activities, (B) hepatic GSH content, (C) plasma BUN levels, (D) renal GSH content, (E) plasma creatinine levels after 24 h, (F) H&E staining of liver and kidney tissues after 24 h. Bars represent means ± SEM for n = 6 mice. *p < 0.05 compared to the APAP-treated group.

To investigate whether APAP-induced nephrotoxicity is primarily the result of direct renal toxicity or secondary to hepatic injury, we used BSO to inhibit γ-glutamyl cysteine synthetase, the rate-limiting enzyme in GSH biosynthesis (Drew and Miners, 1984; Meister, 1988). Previous studies have shown that low-dose BSO pretreatment enhances APAP-induced hepatotoxicity in female mice but not in male mice (Masubuchi et al., 2011), while high-dose BSO pretreatment with 300 mg/kg APAP results in early mortality (Griffith and Meister, 1979; Masubuchi et al., 2011; Miners et al., 1984). To avoid lethality while achieving sufficient GSH depletion, we used a dose of 50 mg/kg BSO. Overnight-fasted male mice were administered 300 mg/kg APAP with or without 50 mg/kg BSO. GSH depletion by BSO exacerbated hepatotoxicity at an earlier time point (3 h) (Fig. 3A) due to a profound decline in hepatic GSH levels (Fig. 3B). APAP administration alone caused an early reduction in hepatic GSH levels at 3 h, but this effect was enhanced in the BSO + APAP group, where GSH levels dropped to undetectable levels at this time point (Fig. 3B). This severe early GSH depletion in the BSO + APAP group correlated with enhanced early hepatocellular injury. Interestingly, despite these initial differences, hepatic GSH levels gradually recovered after 6 h in both treatment groups, reaching control levels by 24 h in the BSO-treated group and even higher levels in the controls (Fig. 3B) with comparable serum ALT levels across all APAP-treated groups at the 24-h timepoint (Fig. 3A). Treatment with BSO alone indicated only a temporary GSH depletion in both organs and no liver or kidney injury (Fig. 5). Mice pretreated with BSO exhibited significantly elevated BUN levels compared to those treated with APAP alone (Fig. 3C). Interestingly, unlike in the liver, renal GSH levels remained severely depleted at 24 h in the BSO + APAP group, indicating that sustained GSH depletion specifically exacerbates APAP-induced kidney injury at this later time point (Fig. 3D). Plasma creatinine, another established marker of kidney function (Kellum et al., 2013; Ostermann et al., 2024), was also significantly elevated in mice treated with APAP + BSO compared to other groups (Fig. 3E). Histopathological analysis of the livers and kidneys confirmed these findings. H&E staining indicated that there was no significant difference in the areas of necrosis in the liver at 24 h (Fig. 3F). However, there was extensive tubular damage in the BSO + APAP group, characterized by pronounced vacuolar degeneration, tubular dilatation, brush border loss, and cast formation (Fig. 3F). This pattern of injury supports a role of GSH depletion in selectively exacerbating APAP-induced kidney damage.

**Fig. 4.**
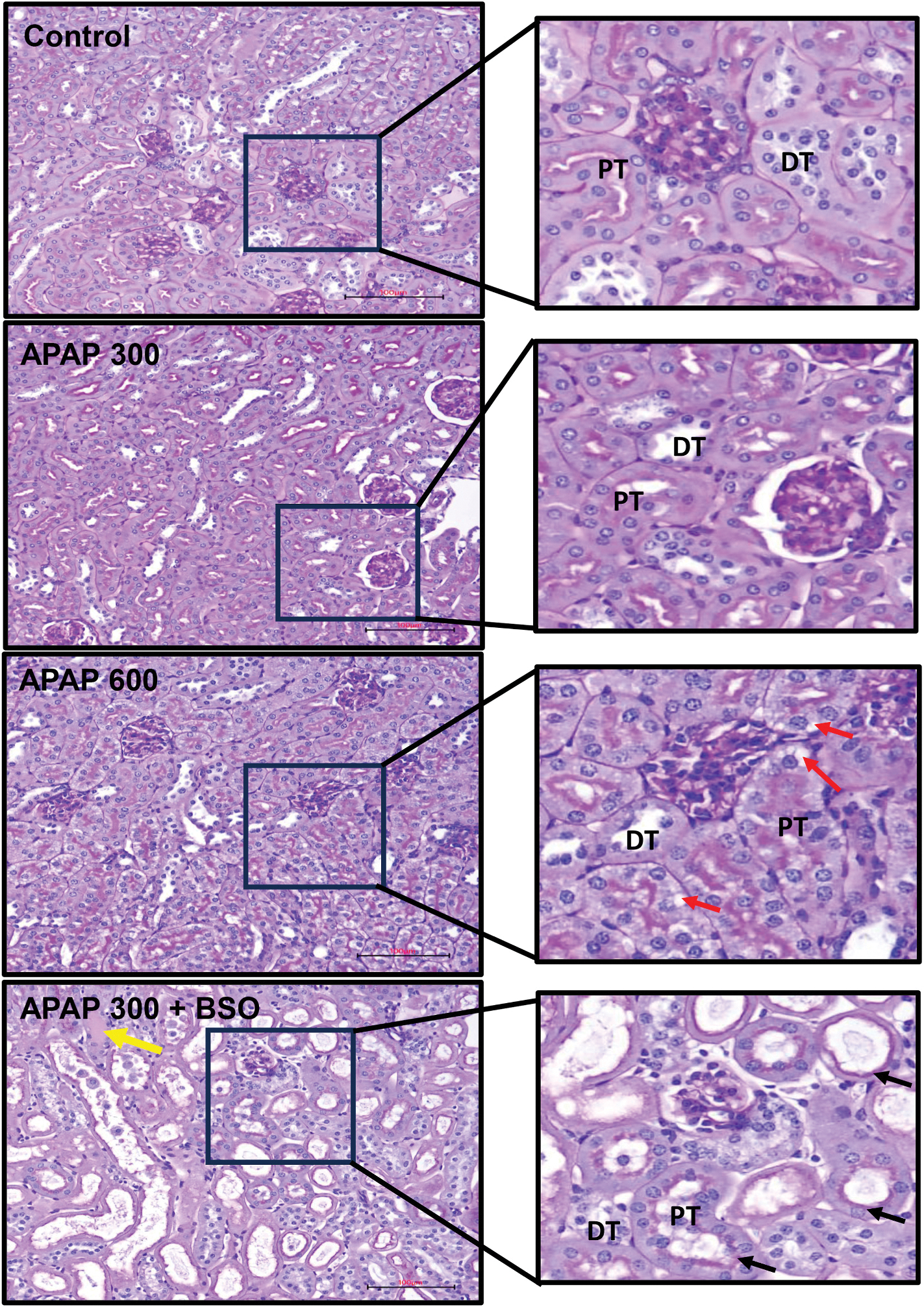
Effects of APAP and BSO on renal histology using PAS staining. Representative PAS-stained kidney sections from an untreated control group or mice treated with 300 mg/kg APAP, 600 mg/kg APAP, and 300 mg/kg APAP with 50 mg/kg BSO. Controls show normal renal histology with intact glomeruli and tubules. In the 300 mg/kg APAP group, normal to mild tubular injury is observed, characterized by vacuolation in a few proximal tubules. In the APAP 600 mg/kg group, there is increased tubular injury, including pronounced vacuolization (red arrows) throughout the cortex. The APAP + BSO group displays moderate to severe tubular damage with extensive vacuolar degeneration and tubular dilatation (black arrows) and cast formation (yellow arrow), indicating exacerbated kidney injury. Scale bars represent 100 μm. These images represent findings from at least five animals per group. (For interpretation of the references to color in this figure legend, the reader is referred to the Web version of this article.)

To assess tubular structural integrity in APAP-induced nephrotoxicity, kidney sections were subjected to Periodic Acid-Schiff (PAS) staining (Fig. 4). This technique was selected for its ability to highlight glycoproteins in proximal tubular brush borders (Gaut and Liapis, 2021; Kellum et al., 2013), enabling clear differentiation between proximal and distal tubules and precise evaluation of structural alterations. PAS staining of kidney sections from vehicle-treated controls revealed normal renal architecture, with intact glomeruli and well-organized proximal tubules (Fig. 4). Mice treated with a dose of 300 mg/kg APAP showed only mild tubular injury, characterized by occasional vacuolization. In contrast, the 600 mg/kg APAP group demonstrated more pronounced tubular damage, with extensive vacuolar degeneration observed throughout the cortex (red arrows). The most severe injury was seen in the 300 mg/kg APAP + BSO group, where PAS staining revealed widespread vacuolar degeneration, tubular dilatation (black arrows), and intraluminal cast formation (yellow arrow). These consistent histopathological findings across multiple animals underscore the exacerbating effect of BSO on APAP-induced renal injury.

### 3.3. Effects of NAC on APAP-induced acute kidney injury

**Fig. 5.**
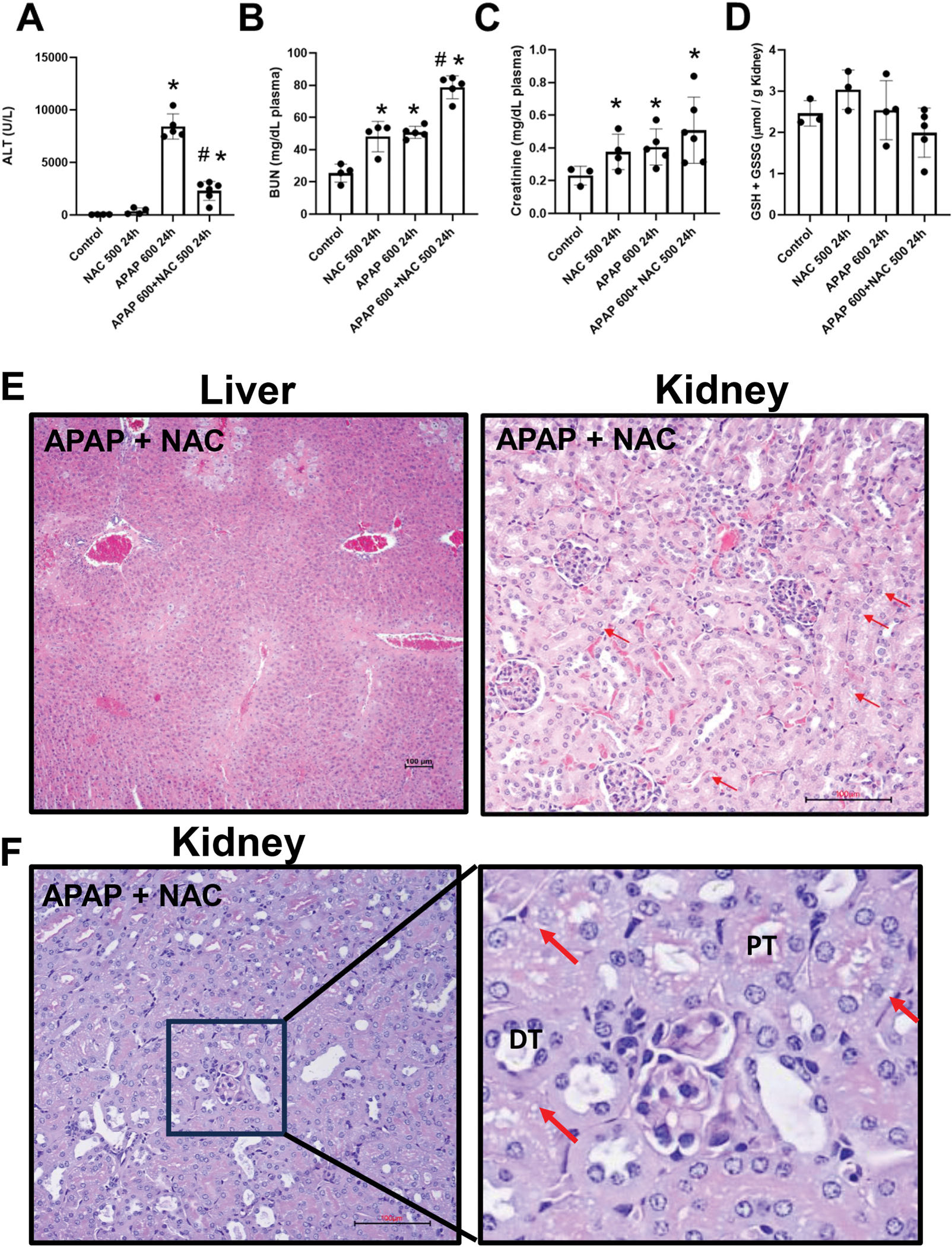
Effect of NAC on APAP-induced liver and kidney injury. Fasted male C57BL/6J mice received a single intraperitoneal injection of APAP (600 mg/kg), either with or without cotreatment with 500 mg/kg NAC, or NAC alone, and were sacrificed at 24 h after APAP. (A) Plasma ALT activities, (B) plasma BUN levels, (C) plasma creatinine levels, (D) renal GSH content, (E) H&E staining of liver and kidney tissues, (F) PAS-stained kidney sections from mice treated with APAP + NAC (red arrows indicate vacuolation). Bars represent means ± SEM for n = 6 mice. *p < 0.05 compared to control; ^#^p < 0.05 compared to APAP-treated groups. (For interpretation of the references to color in this figure legend, the reader is referred to the Web version of this article.)

**Fig. 6.**
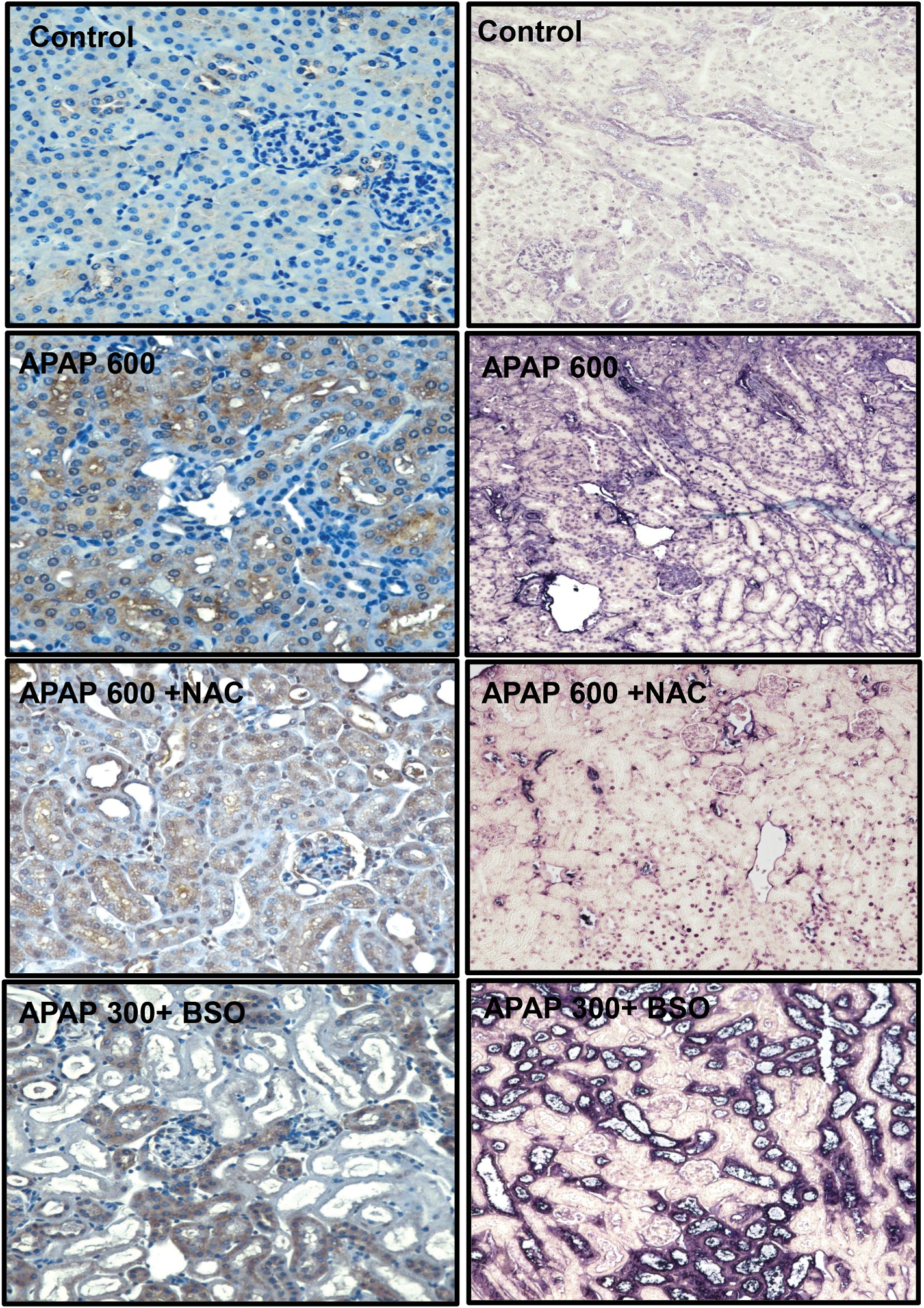
Effects of NAC and BSO on APAP-induced renal injury evaluated by KIM-1 expression and TUNEL staining. Representative immunohistochemistry images of KIM-1 (left, 40x) and TUNEL staining (right, 20x) in kidney sections from male mice across different treatment groups at 24 h after APAP: control, APAP (600 mg/kg), APAP (600 mg/kg) + NAC (500 mg/kg), and APAP (300 mg/kg) + BSO. Images are representative of 5 animals per group.

The data so far demonstrated that sustained GSH depletion with BSO administration significantly exacerbates APAP-induced kidney injury, even as hepatic GSH levels recover (Figs. 3 and 4). This finding suggests a critical role for renal GSH in protecting against APAP nephrotoxicity. Building on these observations, we investigated whether NAC could confirm these findings. While BSO depletes GSH by inhibiting its synthesis, NAC works through an opposite mechanism, i.e., providing cysteine, a rate-limiting precursor for GSH synthesis, thus potentially replenishing GSH stores in damaged tissues (Corcoran et al., 1985; Raghu et al., 2021; Saito et al., 2010). Since NAC is the only FDA-approved antidote for an APAP overdose, this mechanistic contrast offers important insights into organ-specific responses to APAP toxicity and has significant clinical implications for managing APAP overdose. We administered a single intraperitoneal dose of 600 mg/kg APAP to male C57BL/6 J mice, with or without cotreatment of 500 mg/kg NAC, the total dosage equivalent to that used in clinical settings. Tissue and plasma biomarkers were analyzed 24 h post-treatment to evaluate both liver and kidney function (Fig. 5). APAP overdose resulted in a significant elevation of plasma ALT and BUN levels compared to control groups (Fig. 5A and B), confirming the development of both hepatic and renal toxicity. NAC administration significantly attenuated ALT elevation, demonstrating its robust protective effect against liver injury (Fig. 5A). However, despite this hepatoprotection, BUN levels remained significantly elevated in the APAP + NAC group compared to controls, suggesting that protecting the liver does not prevent APAP-induced kidney injury (Fig. 5B). This organ-specific response was further confirmed by plasma creatinine measurements, which remained significantly elevated in the APAP + NAC group compared to controls (Fig. 5C). Interestingly, NAC treatment alone also caused mild BUN and creatinine elevations but no ALT increase, suggesting a limited kidney dysfunction (Fig. 5). Moreover, H&E staining of liver sections from mice treated with APAP + NAC revealed preserved hepatic structure with minimal area of necrosis (Fig. 5E) compared to the extensive necrosis observed in mice treated with the same dose of APAP alone (Fig. 2). This histological improvement correlates strongly with the reduced ALT levels (Fig. 5A) and confirms the potent hepatoprotective effect of NAC. In kidney sections from the APAP + NAC-treated group, H&E staining showed noticeable cytoplasmic vacuolation in proximal tubular cells (Fig. 5E). These vacuolar alterations were more prominently visualized in PAS-stained sections, which revealed persistent and extensive cytoplasmic vacuolation (red arrows). (Fig. 5F). This aligns well with the sustained elevation of renal injury markers (BUN and creatinine), confirming that NAC does not alleviate APAP-induced nephrotoxicity. To assess the extent of kidney injury and cell death, we performed immunohistochemistry for KIM-1, a highly sensitive and specific injury marker for proximal tubular cells (Han et al., 2002), and TUNEL staining. Mice treated with APAP (600 mg/kg) exhibited robust and extensive KIM-1 expression throughout the proximal tubular cells compared to the minimal expression observed in control animals, indicating significant tubular damage (Fig. 6). This was accompanied by widespread TUNEL-positive nuclei (Fig. 6), indicating nuclear fragmentation and apoptotic cell death. Notably, mice co-treated with APAP and NAC demonstrated comparable expression of KIM-1 and patterns and staining intensity of TUNEL-positive cells to the APAP 600 mg/kg group, despite the significant hepatoprotection by NAC. In contrast, mice pretreated with BSO not only exhibited more intense and widespread KIM-1 immunoreactivity throughout the proximal tubules, but TUNEL staining revealed evidence of both apoptotic and necrotic cell death patterns, characterized by cellular swelling and membrane disruption in addition to nuclear fragmentation.

Finally, quantitative histopathological scoring of the renal cortex and outer stripe of the outer medulla (OSOM), regions rich in proximal tubular cells and highly vulnerable to APAP-induced injury (Cummings et al., 2000; Radi, 2019), revealed distinct injury patterns across treatment groups (Table 1). Blinded assessment by a expert renal pathologist (T.A.F.) showed that 300 mg/kg APAP caused minimal renal damage (score 0–1), limited to occasional tubular vacuolation. In contrast, BSO pretreatment markedly worsened injury, producing severe tubular damage (score 4) characterized by extensive dilatation, brush border loss, and prominent cast formation. An APAP dose of 600 mg/kg caused moderate damage (score 2). Importantly, the APAP + NAC treatment group exhibited nearly identical histopathological scores despite the hepatoprotective effect of NAC (Table 1). These findings strongly support the notion that GSH depletion amplifies APAP-induced nephrotoxicity, underscoring the organ-specific nature of APAP toxicity and therapeutic responses, with NAC offering no meaningful renal protection.

## 4. Discussion

### 4.1. The impact of BSO on APAP-induced liver and kidney injury

In an attempt to dissociate liver and kidney injury, animals were treated with BSO, an inhibitor of γ-glutamylcysteine synthetase, the rate-limiting step of cellular GSH synthesis (Drew and Miners, 1984; Meister, 1988). A high dose of 400–800 mg/kg BSO effectively inhibited GSH synthesis in the liver after NAC or GSH administration in fasted mice (Wendel and Jaeschke, 1982) or stem cell injection in APAP-treated mice (Umbaugh et al., 2022) for up to 6 h. However, using a lower dose of 50 mg/kg BSO in the current study, the decline of hepatic GSH levels was slightly accelerated, and the recovery over the next 24 h was only moderately attenuated. This resulted in a minor acceleration of plasma ALT release during the first 3 h after APAP but did not affect longer-term injury at 6–24 h after a 300 mg/kg dose of APAP. In striking contrast to the limited effects in the liver, the 50 mg/kg dose of BSO amplified GSH depletion in the kidney and completely prevented GSH recovery over 24 h. This caused significant kidney injury and dysfunction as demonstrated by increased plasma levels of BUN and creatinine, as well as histological evidence of tubular injury and apoptotic cell death. Importantly, this severe kidney injury after BSO occurred in mice treated with a moderate APAP overdose, which alone does not cause relevant kidney dysfunction. These findings indicate that renal GSH levels are critical for APAP-induced kidney injury, mainly as a scavenger of NAPQI. Hence, in the presence of consistent liver damage, the acute kidney injury and dysfunction can be dramatically modulated. This suggests that both injury processes are independent of each other. Thus, low-level BSO treatment was able to dissociate liver and kidney injury after a moderate APAP overdose.

### 4.2. The impact of NAC treatment on APAP-induced liver and kidney injury

To evaluate if the injury process in the kidney and liver is also independent after a severe APAP overdose that more closely reflects the scenario leading to acute liver failure and death in patients, i.e., an aggravated inflammatory response causing enhanced liver injury (Nguyen et al., 2023) and senescence with impaired regeneration (Bhushan et al., 2014; Umbaugh et al., 2024), NAC treatment was used with the 600 mg/kg APAP dose. Consistent with many previous studies using various doses of APAP and NAC (Corcoran et al., 1985; James et al., 2003; Saito et al., 2010), a single dose of NAC effectively reduced liver injury. NAC restores depleted hepatic GSH (Corcoran and Wong, 1986), which then scavenges NAPQI and prevents protein adduct formation during the metabolism phase (Corcoran et al., 1985), but at later times, GSH is imported into mitochondria and scavenges peroxynitrite (Cover et al., 2005; Saito et al., 2010). In contrast, NAC did not affect renal GSH levels, most likely due to the fact that the early moderate depletion is rapidly restored even in the absence of NAC. Importantly, most parameters of kidney injury and dysfunction, including creatinine, KIM1, TUNEL assay, and histological assessment, did not significantly change in the APAP-treated animals, except BUN levels, which significantly increased. In addition, NAC alone caused a mild increase in BUN and creatinine levels, indicating mild kidney dysfunction. This is consistent with studies showing tubular necrosis with higher doses of NAC (800 mg/kg) through increased oxidant stress and reduced GSH synthesis (Tsai et al., 2024). Furthermore, NAC can also interfere with stress-induced Nrf2 activation and prevent protective adaptive antioxidant responses (Small et al., 2018). These mechanisms, which appear to be more kidney-specific, as even very high doses of NAC are still beneficial in the liver (James et al., 2003), may explain the potential detrimental effects of NAC on kidney function. Taken together, the dramatic reduction of liver injury by NAC had no beneficial effect on kidney injury, which again suggests that APAP-induced liver and kidney injury are independent processes with no indication that injury of one organ directly affects the other.

### 4.3. The contradictive effects of NAC and BSO on APAP-induced liver and kidney injury

Whereas the observation of independent injury processes is consistent with the BSO and NAC experiments, the fact that both interventions target GSH levels but cause different effects on the injury in the liver and kidney appears contradictory. However, the protective effect of NAC in the liver is consistent with the rapid GSH resynthesis when the hepatic GSH pool is stressed due to fasting (Wendel and Jaeschke, 1982) or consumed by conjugation with the reactive metabolite NAPQI (Corcoran et al., 1985; Saito et al., 2010). Hepatic GSH depletion after APAP exposure is very rapid (>90 % depletion within 30 min in the mouse liver), and the spontaneous recovery is dose-dependent (McGill et al., 2013). Administration of NAC or other cysteine donors will result in immediate stimulation of GSH synthesis with recovery of hepatic GSH levels. If that happens during the metabolism phase or the later phase with peroxynitrite formation, the recovery of GSH levels will be protective in mice in vivo (Corcoran et al., 1985; James et al., 2003; Knight et al., 2002) and mouse hepatocytes (Bajt et al., 2004). Similar effects are also observed in human hepatocytes (Xie et al., 2014) and are the basis for the high efficacy of NAC in early-presenting APAP overdose patients (Rumack and Bateman, 2012; Smilkstein et al., 1988). The fact that inhibition of GSH synthesis by BSO did not aggravate the injury in male mice is consistent with a previous publication (Masubuchi et al., 2011). The lack of a significant effect may be explained by the use of a low dose, which only marginally enhanced the already depleted GSH levels and only modestly delayed the recovery. In particular, during the key periods for NAPQI and peroxynitrite scavenging (approximately less than 6 h in the mouse), GSH levels were not affected by BSO, and there was no effect on liver injury.

In contrast to the liver, the renal effects are more difficult to explain. The metabolic activation of APAP in the kidney also involves Cyp2E1 in proximal tubular cells (Akakpo et al., 2020; Hart et al., 1994), leading to the formation of APAP-SG adducts and their metabolites in the outer medulla (Akakpo et al., 2020, 2024). Renal protein adduct formation and kidney injury were prevented by fomepizole (Akakpo et al., 2020), which is a selective inhibitor of Cyp2E1 (Hazai et al., 2002). The detoxification of NAPQI by GSH explains the sensitivity to BSO. The higher responsiveness of the kidney to a low dose of BSO, as compared to the liver, may be caused by higher GSH turnover in the kidney due to the high γ-glutamyl transferase activities requiring a high rate of GSH resynthesis, which may be very sensitive to even moderate inhibition. Nevertheless, the experiment with BSO suggests that renal GSH levels are critical for the defense against APAP toxicity. However, the lack of protection against APAP-induced kidney injury with NAC, as observed in this study and also previously reported (Akakpo et al., 2023; Slitt et al., 2004), appears to contradict these findings. A previously proposed hypothesis is that the APAP-cysteine conjugates may inhibit the γ-glutamyl cycle, leading to decreased renal GSH levels and enhanced injury (Stern et al., 2005a, 2005b). However, renal GSH levels were the same after 24 h, suggesting that there was no inhibition of GSH synthesis. On the other hand, ribose-cysteine treatment enhanced renal GSH levels and protected (Slitt et al., 2005).

An alternative explanation could be that in the renal medulla, prostaglandin endoperoxidase synthetase (PGES) can bioactivate APAP to toxic metabolites such as NAPQI and *p*-benzoquinone (PBQI), resulting in free radical generation and cell death in a region where CYP450 activity is low, indicating a GSH-independent pathway (Mazer and Perrone, 2008; Ściskalska et al., 2015). Additionally, APAP can be metabolized by N-deacetylase, which is highly expressed in the kidney, to form *p*-aminophenol (PAP). This metabolite can covalently bind to protein sulfhydryl groups, leading to oxidative stress and nephrotoxicity independent of GSH status (Tsai et al., 2024). Investigating each of these potential mechanisms represents important directions for future research.

### 4.4. Summary and conclusions

Using 2 different approaches, inhibition of GSH synthesis with BSO and supply of the rate-limiting amino acid cysteine with NAC, we could modulate both APAP-induced liver and kidney injury. In the case of a low dose of BSO, significant kidney injury was induced after a moderate APAP overdose, while at the same time, liver injury was not significantly affected. On the other hand, NAC treatment effectively reduced liver injury after a severe APAP overdose without attenuating the substantial kidney injury. Thus, the modulation of the injury in one organ without impact on the other suggests that the injury processes are independent of each other. A limitation of this study is the relatively short observation window, which restricted our ability to evaluate delayed renal injury or recovery beyond 24 h. Extending the time course was not feasible, particularly for the APAP-alone group, as severe hepatic failure leads to high mortality beyond this time point. Moreover, while the mechanisms of APAP-induced liver injury are well characterized, the underlying pathways contributing to APAP-induced AKI remain comparatively less studied. Future studies designed to extend the observation period will be valuable in elucidating the delayed and mechanistic aspects of APAP-induced nephrotoxicity. The obvious differences in APAP-induced liver versus kidney injury mean that organ-specific interventions are needed, or a therapeutic target common to both mechanisms, e.g., Cyp2E1 inhibition by fomepizole, needs to be pursued. However, the fact that the injury mechanisms in the liver and kidney are independent does not mean that one, i.e., AKI, is not important for the overall outcome. In very severe overdose patients, acute liver failure may develop into multiple organ failure and death, with the kidney playing a critical part in the negative outcome (Antoine et al., 2015).

## Figures and Tables

**Table 1:** Blinded scoring of APAP-induced acute kidney injury.

		Histopathology	Scores	
				
Group	0–1	2	3	4

APAP 300	5	0	0	0
APAP 300 + BSO	0	0	0	5
APAP 600	1	5	0	0
APAP 600 + NAC	1	4	0	1

Scoring criteria based on injury severity and affected tissue percentage for each group.

Score 1: Mild injury affecting <10 % of the cortex.

Score 2: Mild injury affecting >50 % of the cortex.

Score 3: Moderate-severe injury affecting <10 % of the cortex.

Score 4: Moderate-severe injury affecting >50 % of the cortex.

Numbers below each score indicate the count of mice scored within each range.

## Data Availability

No data was used for the research described in the article.
